# Root Canal Therapy Versus Single-Tooth Dental Implants for Compromised Teeth: A Retrospective Comparative Study

**DOI:** 10.7759/cureus.115446

**Published:** 2026-08-30

**Authors:** Aravind A Vijayan, Piyali Sarkar, Bhogadi V Madhuri, A. Vinod Kumar, Ankit Mohapatra, Ridyumna Garain, Md Kafeel Ahmed

**Affiliations:** 1 Dentistry, Mount Zion Medical College, Adoor, IND; 2 Prosthodontics and Crown and Bridge, Mithila Minority Dental College and Hospital, Darbhanga, IND; 3 Conservative Dentistry and Endodontics, Sri Sai College of Dental Surgery, Vikarabad, IND; 4 Conservative Dentistry and Endodontics, Meghna Institute of Dental Sciences, Nizamabad, IND; 5 Public Health Dentistry, Hi-Tech Dental College and Hospital, Bhubaneswar, IND; 6 Conservative Dentistry and Endodontics, Army Dental Corps, Mumbai, IND; 7 Periodontology, MNR Dental College and Hospital, Sangareddy, IND

**Keywords:** compromised teeth, dental implants, endodontic treatment, patient satisfaction, root canal therapy, treatment outcomes

## Abstract

Background: Selecting between root canal therapy (RCT) and extraction followed by dental implant placement for a compromised tooth remains clinically challenging. Treatment decisions should consider survival, complications, treatment burden, cost, and patient satisfaction rather than survival alone.

Objective: To compare treatment survival, clinical and radiographic success, complications, treatment visits, duration, direct cost, and patient satisfaction between RCT-restored teeth and single implant-supported crowns.

Materials and methods: This retrospective comparative cohort study included 100 patients, comprising 50 patients treated with RCT, followed by definitive restoration, and 50 patients treated with extraction, followed by single implant-supported crown rehabilitation, each with a minimum follow-up of 12 months. Demographic, clinical, radiographic, complication-related, economic, and patient-satisfaction data were evaluated. Categorical variables were compared using the chi-square or Fisher's exact test, continuous variables using the independent-samples t-test, and the ordinal satisfaction score using the Mann-Whitney U test. Effect estimates are reported with 95% confidence intervals (CIs). Statistical significance was set at p < 0.05.

Results: Treatment survival was 100% following RCT and 96% following implant treatment (risk difference: 4.0%, 95% CI: −3.7 to 13.5; p = 0.495). Clinical and radiographic success did not differ significantly. Biological complications were less frequent after RCT than after implant treatment (4% versus 20%; risk difference: −16.0%, 95% CI: −29.4 to −3.1; Fisher's exact test, p = 0.028). RCT was associated with fewer visits (mean difference: −2.12, 95% CI: −2.47 to −1.77), a shorter treatment duration (−87.10 days, 95% CI: −96.57 to −77.63), and a lower direct cost (−INR: 29,966, 95% CI: −32,203 to −27,729); all p < 0.001. Documented patient satisfaction scores were higher in the RCT group than in the implant group (median: 5 (IQR: 4-5) versus 4 (IQR: 4-5); p = 0.012, Mann-Whitney U test).

Conclusions: Both treatments demonstrated favorable short- to intermediate-term outcomes. In this retrospective cohort, RCT was associated with fewer biological complications, lower treatment burden and cost, and higher satisfaction. Because treatment was not randomized, these associations may partly reflect treatment-selection differences, and natural-tooth preservation should be considered when a compromised tooth remains predictably restorable.

## Introduction

A compromised tooth can present with extensive caries, pulpal disease, periapical disease, extensive structural loss, fracture, previous endodontic treatment failure, and/or diminished periodontal support [1]. A detailed evaluation of restorability, remaining coronal tissue, ferrule space, periodontal status, crown-to-root ratio, periapical status, strategic position in the dental arch, and overall patient health must all be taken into account in its management [1]. The main clinical question is whether to preserve the natural tooth with root canal therapy (RCT) and a definitive restoration or to extract it and replace it with a single implant-supported crown [2].

RCT involves removal of inflamed or infected pulpal tissue, disinfection and sealing of the root canal system, and promotion of periapical healing [3]. When combined with a suitable coronal reconstruction, it allows maintenance of a natural tooth together with its periodontal ligament, proprioception, and surrounding alveolar tissues [4]. Appropriate definitive coronal restorations, including adhesive endocrowns and full-coverage crowns, are important for protecting the remaining tooth structure and maintaining the clinical success of extensively restored teeth [5]. Factors that affect its long-term outcome include the severity of periapical disease before treatment, the quality of canal preparation and obturation, the remaining tooth structure, the presence of a ferrule, periodontal support, and integrity of the final restoration [1]. With current advances in magnification, instrumentation, irrigation, biomaterials, and endodontic microsurgery, the prognosis of a tooth that may previously have been considered questionable has improved [3]. Nevertheless, a risk of recurrent infection, coronal leakage, root fracture, and restorative failure remains [4,6].

Tooth extraction followed by implant placement is a well-established replacement option when a tooth is not restorable or has an unfavorable endodontic, structural, or periodontal prognosis [7]. Although adequate bone and soft tissue conditions, osseointegration, and prosthetic rehabilitation are required, an implant-supported crown restores function and esthetics without preparation of the adjacent teeth [7]. In some cases, socket preservation, bone augmentation, or soft-tissue grafting may be needed, lengthening treatment time, cost, and morbidity [8]. Dental implants can also experience biological and technical complications, such as peri-implant mucositis, peri-implantitis, marginal bone loss, screw loosening, and crown fracture [9].

Direct comparison of these two treatment approaches remains difficult because success and survival are not always reported consistently across endodontic and implant studies. Additional factors - including restorability, periodontal prognosis, bone availability, systemic health, smoking, esthetic expectations, clinician expertise, patient preference, cost, and anticipated treatment duration - influence treatment selection [2]. At present, no evidence indicates that either method is universally superior; both can achieve high survival rates when correctly indicated, but with distinct differences in their failure and complication profiles [7]. Previous reports have focused mainly on survival, with less attention to clinical and radiographic success, complications, additional interventions, treatment visits, duration, cost, and patient satisfaction [2,10]. For this reason, the present study compared the clinical, radiographic, economic, and patient satisfaction outcomes of RCT and single-implant-supported crowns to support individualized, patient-centered treatment decisions for compromised teeth [10,11].

Therefore, this study aimed to compare the clinical and patient-centered outcomes of RCT, followed by definitive restoration versus single-tooth dental implant treatment for compromised teeth, including treatment survival, clinical and radiographic success, biological and technical complications, treatment visits, treatment duration, direct cost, and patient satisfaction.

## Materials and methods

Study design and setting

This retrospective comparative cohort study was conducted at a tertiary dental care center involving the departments of Conservative Dentistry and Endodontics, Prosthodontics, and Oral Implantology. Clinical and radiographic records of patients who underwent RCT followed by definitive restoration, or extraction followed by single-tooth implant rehabilitation, were reviewed. The study was reported in accordance with the Strengthening the Reporting of Observational Studies in Epidemiology (STROBE) guidelines for cohort studies [12].

Study Period and Follow-Up

Patients who underwent treatment at Mount Zion Medical College, Chayalode, Adoor, India, between April 2023 and September 2024 were included in the study. Follow-up data were reviewed after completion of definitive restoration, and only records with a minimum follow-up of 12 months were included to permit evaluation of treatment survival, clinical success, radiographic findings, complications, and documented patient satisfaction.

Ethical Considerations

In accordance with the institutional policy governing retrospective studies based exclusively on pre-existing, de-identified clinical records, ethical clearance was not required for this study. The study involved no direct patient contact or intervention, and all data were de-identified before analysis and handled confidentially in accordance with applicable institutional policies and ethical principles. Individual informed consent was not required in accordance with institutional policy because the study involved retrospective review of pre-existing, de-identified clinical records without direct patient contact or intervention.

Study population and sample size

The study population comprised adult patients with a single compromised permanent tooth managed either by RCT followed by definitive coronal restoration or by extraction followed by placement of a single implant-supported crown. All consecutive eligible records meeting the predefined inclusion and exclusion criteria during the study period were considered for inclusion. Tooth prognosis was classified as favorable, questionable, or compromised according to the clinical assessment documented in the treating clinician's records. Because this was a retrospective study, the specific clinical criteria underlying these classifications were not consistently documented and could not be retrospectively standardized. A total of 100 eligible records were identified, comprising 50 patients in the RCT group and 50 patients in the dental implant group. No formal a priori sample-size calculation was performed; instead, all available eligible records during the study period were included.

Participant Selection

Patient records were identified from clinical registers and electronic or paper-based databases and screened according to predetermined eligibility criteria. Consecutive eligible records were included to reduce selection bias. Demographic characteristics, baseline clinical condition, treatment details, radiographic findings, treatment survival, complications, number of visits, treatment duration, direct cost, and patient satisfaction were recorded using a standardized data-collection form. Participants were categorized according to the treatment received.

Inclusion Criteria

Patients aged 18 years or older; treatment of a single compromised permanent tooth; completion of RCT with a definitive restoration, or extraction followed by a single implant-supported crown; availability of complete baseline and follow-up clinical and radiographic records; a functional definitive restoration; and a minimum follow-up period of 12 months were included.

Exclusion Criteria

Patients with incomplete clinical or radiographic records, follow-up shorter than 12 months, uncontrolled systemic disease, untreated advanced periodontitis, multiple adjacent missing teeth, implant-supported bridges, confirmed vertical root fracture at initial assessment, complex trauma-related defects, or incomplete definitive restoration were excluded.

Study Groups

Eligible participants were categorized into two groups according to the treatment received. Group I consisted of patients whose compromised teeth were managed by RCT followed by definitive coronal restoration. Group II consisted of patients who underwent extraction of the compromised tooth followed by placement of a single dental implant-supported crown. Each group included 50 patients.

Baseline clinical and radiographic assessment

Baseline assessment included age, sex, relevant medical and dental history, smoking status, diabetes status, tooth type, dental arch, periodontal condition, remaining coronal tooth structure, mobility, restorability, periapical status, and initial prognosis. Preoperative intraoral periapical radiographs were examined to assess caries extent, root morphology, periapical lesions, periodontal bone support, and the condition of previous restorations or endodontic treatment. In the implant group, available bone height and width and anatomical limitations at the proposed implant site were also evaluated. Radiographic assessments were based on the radiographs available in the clinical records. Because the study was retrospective, examiner calibration, blinding, and formal assessment of inter- or intra-examiner reliability were not performed.

Treatment Procedures

Treatments were performed by qualified dental professionals according to routine institutional clinical protocols. Where documented in the available records, details of instrumentation systems, irrigation protocols, obturation materials, implant systems, surgical procedures, and restorative materials were extracted. However, these details were not consistently available for all patients because of the retrospective nature of the study.

RCT and Definitive Restoration

Following administration of local anesthesia and rubber-dam isolation, an access cavity was prepared, and the root canals were located. Working length was determined using an electronic apex locator and confirmed radiographically when required. The canals were cleaned and shaped using rotary or hand instrumentation with appropriate irrigation. After adequate disinfection and drying, the canals were obturated using gutta-percha and a root canal sealer. The access cavity was sealed, and the tooth subsequently received an appropriate definitive restoration - a direct restoration, post-and-core restoration, or full-coverage crown - depending on the remaining tooth structure and restorative requirements. Specific instrumentation systems, irrigant types or concentrations, and obturation systems were not consistently documented in the available records and therefore could not be reliably reported

Extraction, Implant Placement, and Prosthetic Restoration

The compromised tooth was extracted using an atraumatic technique under local anesthesia. Implant placement was performed either immediately or after healing, depending on the clinical condition of the extraction socket and available bone. Bone augmentation or socket-preservation procedures were performed when clinically indicated. After implant placement and an appropriate osseointegration period, the implant was restored with a single crown. Occlusion, proximal contacts, peri-implant soft tissues, stability, and radiographic bone levels were assessed after prosthetic restoration. Where documented in the available records, implant system and dimensions, loading protocols, augmentation procedures, and prosthetic materials and protocols were extracted. These details were not consistently available across all records because of the retrospective nature of the study.

Outcome Measures

The primary outcome was treatment survival at final follow-up, defined as the root canal-treated tooth or dental implant remaining present, functional, and capable of supporting its definitive restoration without extraction or implant removal. Secondary outcomes included clinical and radiographic success, biological and technical complications, need for additional intervention, number of treatment visits, total treatment duration, direct treatment cost, and patient satisfaction.

Treatment duration was calculated as the interval from the first treatment visit to placement of the definitive restoration and, in the implant group, included the healing and osseointegration period. Direct treatment cost was defined as the total institutional charge associated with the treatment episode, including the relevant procedural, restorative or prosthetic, radiographic, implant-related, and adjunctive procedures where applicable. Patient satisfaction was obtained from routinely documented post-treatment records using the available five-point satisfaction scale. Because the scale was retrospectively extracted from existing records, its original development, formal validation, timing of administration, and completeness of administration could not be independently verified.

Criteria for Treatment Success and Failure

The clinical success criteria were treatment-specific, reflecting the distinct biological and mechanical failure modes of root canal-treated teeth and implants; direct equivalence between the two endpoints should therefore not be assumed. RCT was considered clinically successful when the tooth remained functional and demonstrated no persistent pain, swelling, sinus tract, pathological mobility, tenderness, or progressive periapical disease. Implant treatment was considered clinically successful when the implant remained stable and functional without persistent pain, suppuration, pathological mobility, progressive marginal bone loss, or uncontrolled peri-implant disease.

Radiographic success was assessed on periapical radiographs at final follow-up. For root canal-treated teeth, it was defined as absence or reduction of periapical radiolucency with no new or progressive periapical disease; for implants, it was defined as the absence of progressive or pathological peri-implant marginal bone loss. A validated radiographic scoring system (e.g., the periapical index) was not applied retrospectively, which is acknowledged as a limitation.

Biological complications (recurrent periapical infection, caries, periodontal disease, peri-implant mucositis, and peri-implantitis) and technical complications (tooth or restoration fracture, loss of crown retention, screw loosening, and prosthetic fracture) were analyzed as predefined composite outcomes. Individual component events were not consistently retrievable from the retrospective records and were therefore not analyzed separately. Treatment failure was defined as extraction of the root canal-treated tooth or removal of the dental implant. The clinical and radiographic criteria were based on treatment-specific findings documented in the available records. Because of the retrospective design, a uniform validated scoring system could not be applied retrospectively across both treatment groups.

Data Collection Procedure

Data were extracted from clinical records, radiographs, treatment registers, billing records, and follow-up assessments using a standardized data collection form. Each participant was assigned a unique study identification number, and no personally identifiable information was recorded. Data were entered into an electronic spreadsheet and checked for completeness, consistency, duplicate entries, and out-of-range values before statistical analysis. Records with missing information for a specific variable were excluded from that particular analysis, and no statistical imputation was performed.

Statistical analysis

Categorical variables were summarized as frequencies and percentages, and continuous variables as mean and standard deviation. Categorical variables were compared using the Pearson chi-square test or Fisher's exact test, as appropriate. Fisher's exact test was used for comparisons involving sparse outcome events. The independent-samples t-test was used to compare mean treatment visits, treatment duration, and direct cost. The five-point patient-satisfaction score was treated as an ordinal variable and compared using the Mann-Whitney U test, with results expressed as median and interquartile range (IQR); the mean and standard deviation are also presented descriptively. For the principal outcomes, effect estimates risk differences for binary outcomes and mean differences for continuous outcomes are reported with 95% confidence intervals (CIs). Because only 12 biological complications occurred in total, a multivariable regression model was considered likely to be statistically unstable and was therefore not undertaken. Potential confounding was instead assessed through comparison of baseline characteristics and explicitly acknowledged as a limitation, with residual and unmeasured confounding remaining possible. All tests were two-sided, and a p-value less than 0.05 was considered statistically significant.

## Results

Participant selection and follow-up

After record screening and application of the eligibility criteria, 100 participants were included in the final analysis, equally divided between the RCT group (n = 50) and the dental implant group (n = 50). All 100 included records had complete data for the variables included in the final analyses; therefore, no observations were excluded because of missing outcome data. The mean follow-up duration was 23.02 ± 7.34 months in the RCT group and 23.64 ± 6.47 months in the implant group, with no significant difference (p = 0.655). The participant-selection and final-analysis process is presented in Figure 1.

**Figure 1:**
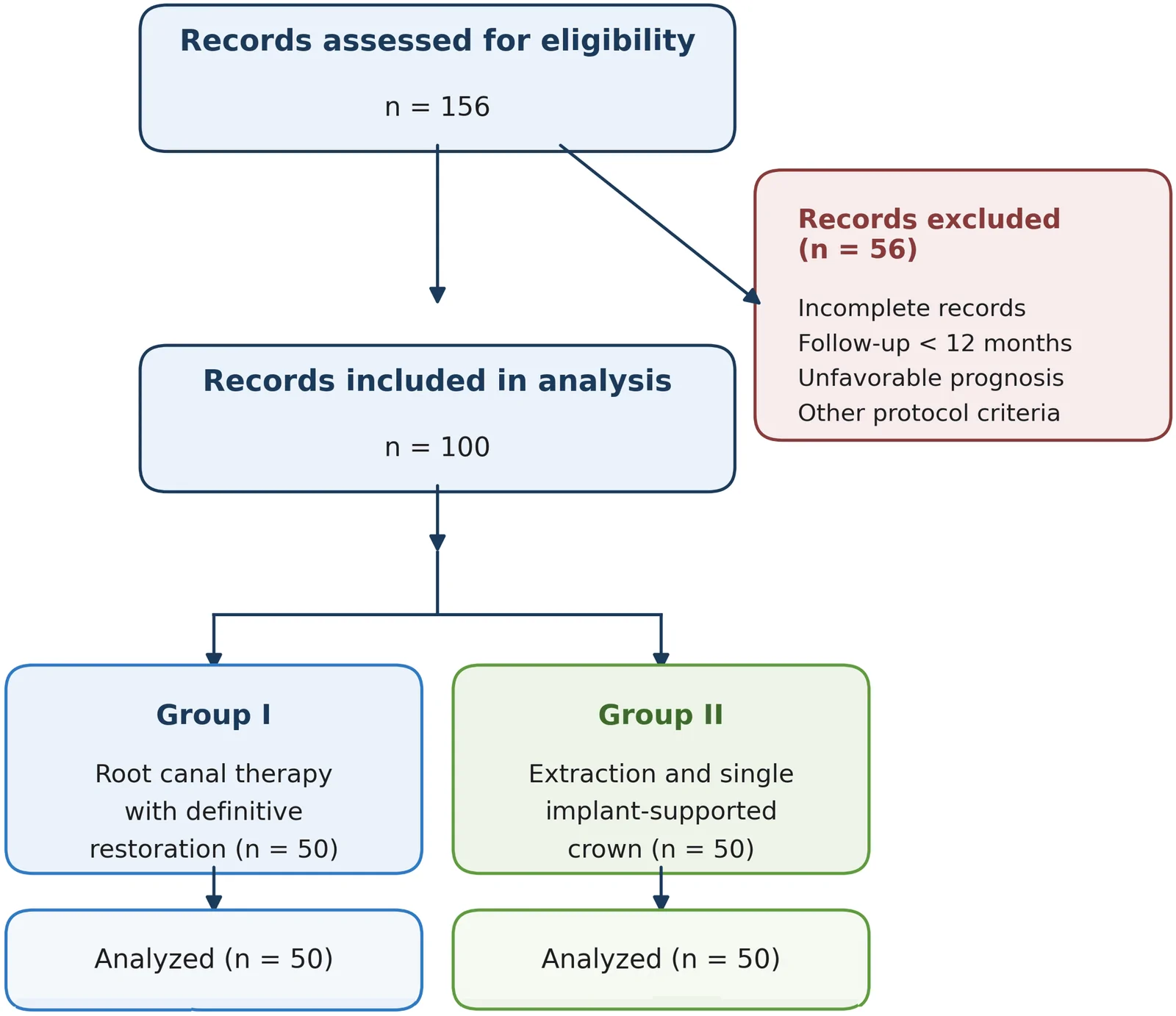
Flow diagram of participant selection and final analysis

Demographic and baseline clinical characteristics

The mean age was 42.46 ± 10.11 years in the RCT group and 46.74 ± 11.53 years in the implant group (independent-samples t-test, t{98} = −1.97, p = 0.051). The groups were broadly similar with respect to demographic and baseline clinical characteristics, including sex, tooth type, dental arch, current smoking, diabetes, baseline prognosis, and follow-up duration (all χ² or t tests, p > 0.05; see Table 1 for individual test statistics); however, periapical lesions were significantly more frequent in the RCT group than in the implant group (32 of 50 (64.0%) versus 22 of 50 (44.0%); Pearson chi-square test, χ²(1) = 4.03, p = 0.045), a baseline imbalance of potential clinical relevance. Molar teeth were the most frequently treated teeth, accounting for 21 of 50 cases (42.0%) in each group. Baseline comparisons, including test statistics for every characteristic, are summarized in Table 1.

**Table 1:** Comparison of demographic and baseline clinical characteristics between the root canal therapy and dental implant groups Values are presented as n (%) unless otherwise indicated. Test statistics are reported as t-statistics for independent-samples t-tests and χ²-statistics for Pearson chi-square tests. A p-value < 0.05 was considered statistically significant. RCT: root canal therapy; SD: standard deviation

Characteristic	Category/statistic	RCT group (n = 50)	Implant group (n = 50)	Statistical test	Test statistic	p-value
Age (years)	Mean ± SD	42.46 ± 10.11	46.74 ± 11.53	Independent-samples t-test	t(98) = −1.97	0.051
Sex	Female	22 (44.0%)	29 (58.0%)	Pearson chi-square	χ²(1) = 1.96	0.161
	Male	28 (56.0%)	21 (42.0%)			
Tooth type	Incisor	5 (10.0%)	7 (14.0%)	Pearson chi-square	χ²(3) = 0.58	0.9
	Canine	9 (18.0%)	7 (14.0%)			
	Premolar	15 (30.0%)	15 (30.0%)			
	Molar	21 (42.0%)	21 (42.0%)			
Dental arch	Maxillary	30 (60.0%)	22 (44.0%)	Pearson chi-square	χ²(1) = 2.56	0.109
	Mandibular	20 (40.0%)	28 (56.0%)			
Current smoker	Yes	6 (12.0%)	5 (10.0%)	Pearson chi-square	χ²(1) = 0.10	0.749
	No	44 (88.0%)	45 (90.0%)			
Diabetes	Yes	7 (14.0%)	7 (14.0%)	Pearson chi-square	χ²(1) = 0.00	1
	No	43 (86.0%)	43 (86.0%)			
Baseline prognosis	Favorable	35 (70.0%)	31 (62.0%)	Pearson chi-square	χ²(1) = 0.71	0.398
	Questionable	15 (30.0%)	19 (38.0%)			
Periapical lesion	Yes	32 (64.0%)	22 (44.0%)	Pearson chi-square	χ²(1) = 4.03	0.045
	No	18 (36.0%)	28 (56.0%)			
Follow-up (months)	Mean ± SD	23.02 ± 7.34	23.64 ± 6.47	Independent-samples t-test	t(98) = −0.45	0.655

Treatment survival and clinical success

Treatment survival at final follow-up was 50 of 50 (100.0%) in the RCT group and 48 of 50 (96.0%) in the implant group (risk difference: 4.0%; 95% CI: −3.7 to 13.5; Fisher's exact test, p = 0.495). Clinical success was achieved in 47 of 50 RCT cases (94.0%) and 46 of 50 implant cases (92.0%) (risk difference: 2.0%; 95% CI: −9.3 to 13.5; Fisher's exact test, p = 1.000). Fisher's exact test does not yield a test statistic analogous to χ²; it was used for both comparisons because of small expected cell counts. Radiographic success was numerically higher after implant treatment than after RCT (45 of 50 (90.0%) versus 39 of 50 (78.0%); risk difference: −12.0%; 95% CI: −26.4 to 2.6; Pearson chi-square test, χ²(1) = 2.68, p = 0.102). These findings should be interpreted cautiously because treatment-specific radiographic criteria were used and a validated radiographic scoring system was not applied retrospectively. Comparative survival and clinical and radiographic outcomes, with test statistics, are shown in Table 2.

**Table 2:** Comparison of treatment survival and clinical and radiographic success between the root canal therapy and dental implant groups Risk difference is calculated as the RCT proportion minus the implant proportion (Newcombe method). †Fisher's exact test is computed directly from the hypergeometric distribution and does not produce a companion test statistic; it was used in place of the chi-square test because of small expected cell counts. A p-value < 0.05 was considered statistically significant. CI: confidence interval; N/A: not applicable; RCT: root canal therapy

Treatment outcome	RCT group, n (%)	Implant group, n (%)	Risk difference, % (95% CI)	Statistical test	Test statistic	p-value
Treatment survival	50 (100.0%)	48 (96.0%)	4.0 (−3.7 to 13.5)	Fisher’s exact test	N/A†	0.495
Clinical success	47 (94.0%)	46 (92.0%)	2.0 (−9.3 to 13.5)	Fisher’s exact test	N/A†	1
Radiographic success	39 (78.0%)	45 (90.0%)	−12.0 (−26.4 to 2.6)	Pearson chi-square	χ²(1) = 2.68	0.102

Biological and technical complications

Biological complications were less frequent in the RCT group than in the implant group (2 of 50 (4.0%) versus 10 of 50 (20.0%); risk difference: −16.0%, 95% CI: −29.4 to −3.1; odds ratio: 0.17, 95% CI: 0.03-0.81; Fisher's exact test, p = 0.028; Fisher's exact test does not produce a companion test statistic). Technical complications occurred in four of 50 RCT cases (8.0%) and six of 50 implant cases (12.0%) (risk difference: −4.0%; 95% CI: −16.8 to 8.6; Pearson chi-square test, χ²(1) = 0.44, p = 0.505). Additional interventions were required in 4 of 50 patients (8.0%) after RCT and 9 of 50 patients (18.0%) after implant treatment (risk difference: −10.0%; 95% CI: −23.7 to 3.6; Pearson chi-square test, χ²(1) = 2.21, p = 0.137). Complication and additional-intervention findings, with test statistics, are provided in Table 3.

**Table 3:** Comparison of biological and technical complications and additional interventions between the treatment groups Biological and technical complications were analyzed as predefined composite outcomes. Risk difference is the RCT proportion minus the implant proportion (Newcombe method). Fisher's exact test was used for the biological complication comparison because of the sparse event count. †Fisher's exact test does not produce a companion test statistic. A p-value < 0.05 was considered statistically significant. CI: confidence interval; N/A: not applicable; RCT: root canal therapy

Complication or intervention	RCT group, n (%)	Implant group, n (%)	Risk difference, % (95% CI)	Statistical test	Test statistic	p-value
Biological complication	2 (4.0%)	10 (20.0%)	−16.0 (−29.4 to −3.1)	Fisher’s exact test	N/A†	0.028
Technical complication	4 (8.0%)	6 (12.0%)	−4.0 (−16.8 to 8.6)	Pearson chi-square	χ²(1) = 0.44	0.505
Additional intervention required	4 (8.0%)	9 (18.0%)	−10.0 (−23.7 to 3.6)	Pearson chi-square	χ²(1) = 2.21	0.137

Treatment visits, duration, and cost

RCT was associated with significantly fewer clinical visits than implant treatment (2.96 ± 0.73 versus 5.08 ± 0.99 visits; mean difference: −2.12, 95% CI: −2.47 to −1.77; independent-samples t-test, t{98} = −12.23, p < 0.001). The mean treatment duration was significantly shorter for RCT than for implant rehabilitation (21.66 ± 9.45 versus 108.76 ± 32.08 days; mean difference: −87.10 days, 95% CI: −96.57 to −77.63; t{98} = −18.42, p < 0.001). The mean direct treatment cost was lower for RCT than for implant treatment (INR 14,696 ± 2,926 versus INR 44,662 ± 7,357; mean difference: −INR 29,966, 95% CI: −32,203 to −27,729; t{98} = −26.76, p < 0.001). These treatment-burden comparisons, with full test statistics, are reported in Table 4.

**Table 4:** Comparison of treatment visits, treatment duration, and direct treatment cost between the root canal therapy and dental implant groups Comparisons were performed using the independent-samples t-test (equal sample sizes, df = 98). Mean difference is the RCT mean minus the implant mean. A p-value < 0.05 was considered statistically significant. CI: confidence interval; INR: Indian rupee; RCT: root canal therapy; SD: standard deviation

Treatment-related outcome	RCT group (mean ± SD)	Implant group (mean ± SD)	Mean difference (95% CI)	Statistical test	Test statistic	p-value
Number of treatment visits	2.96 ± 0.73	5.08 ± 0.99	−2.12 (−2.47 to −1.77)	Independent-samples t-test	t(98) = −12.23	< 0.001
Treatment duration (days)	21.66 ± 9.45	108.76 ± 32.08	−87.10 (−96.57 to −77.63)	Independent-samples t-test	t(98) = −18.42	< 0.001
Direct treatment cost (INR)	14,696 ± 2,926	44,662 ± 7,357	−29,966 (−32,203 to −27,729)	Independent-samples t-test	t(98) = −26.76	< 0.001

Patient satisfaction

Patient satisfaction was favorable in both groups but significantly higher after RCT. The median satisfaction score was 5 (IQR: 4-5) in the RCT group and 4 (IQR: 4-5) in the implant group (Mann-Whitney U test, U = 1585.5, z = 2.51, p = 0.012); the corresponding mean scores were 4.48 ± 0.68 and 4.10 ± 0.79 (mean difference: 0.38 points; 95% CI: 0.09-0.67). The highest score of 5 was reported by 29 of 50 RCT participants (58.0%) and 17 of 50 implant participants (34.0%), and a score of 4 by 16 of 50 (32.0%) and 22 of 50 (44.0%), respectively. No participant reported a score of 1 (0 of 50 (0.0%) in each group). Patient-satisfaction findings, with the full test statistic, are presented in Table 5.

**Table 5:** Comparison of patient satisfaction between the root canal therapy and dental implant groups Patient satisfaction was measured on a five-point ordinal scale (1 = very dissatisfied; 5 = very satisfied) and compared using the Mann-Whitney U test (U = 1585.5, z = 2.51, normal approximation with tie correction). The mean difference was 0.38 points (95% CI: 0.09-0.67). A p-value < 0.05 was considered statistically significant. CI: confidence interval; IQR: interquartile range; RCT: root canal therapy; SD: standard deviation

Patient satisfaction measure	RCT group (n = 50)	Implant group (n = 50)	Test statistic	p-value
Median (IQR)	5 (4-5)	4 (4-5)	U = 1585.5; z = 2.51	0.012
Mean ± SD	4.48 ± 0.68	4.10 ± 0.79	—	—
Score 1, n (%)	0 (0.0%)	0 (0.0%)	—	—
Score 2, n (%)	0 (0.0%)	1 (2.0%)	—	—
Score 3, n (%)	5 (10.0%)	10 (20.0%)	—	—
Score 4, n (%)	16 (32.0%)	22 (44.0%)	—	—
Score 5, n (%)	29 (58.0%)	17 (34.0%)	—	—

Overall comparative outcomes

Overall, both RCT and implant-supported rehabilitation demonstrated high treatment survival and clinical success. No significant differences were identified in survival, clinical success, radiographic success, technical complications, or additional interventions. RCT was associated with significantly fewer biological complications, fewer clinical visits, shorter treatment duration, lower direct cost, and higher patient satisfaction. Implant treatment showed numerically higher radiographic success, although this difference was not statistically significant. These associations should be interpreted alongside treatment burden, complications, cost, and patient preferences when selecting treatment for compromised teeth. The principal comparative outcomes are illustrated in Figure 2.

**Figure 2:**
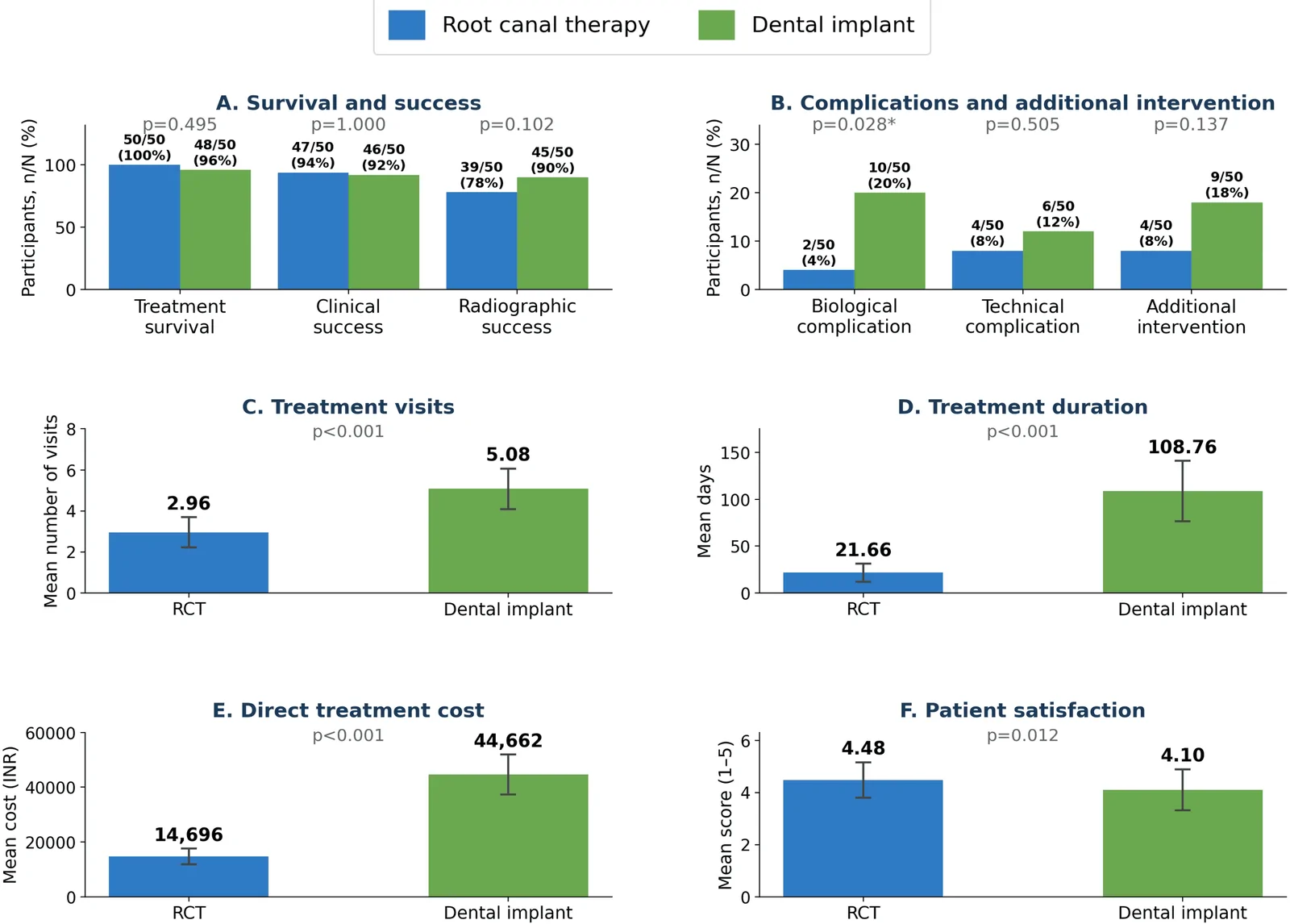
Principal comparative outcomes between the root canal therapy and dental implant groups Panels A and B show outcomes as n/N (%); panels C-F show mean ± SD. Panel A: treatment survival, clinical success, and radiographic success. Panel B: biological complications, technical complications, and additional interventions. Fisher's exact test was used for treatment survival, clinical success, and biological complications; Pearson chi-square test was used for radiographic success (χ²(1) = 2.68, p = 0.102), technical complications (χ²(1) = 0.44, p = 0.505), and additional interventions (χ²(1) = 2.21, p = 0.137). Independent-samples t-tests were used for panels C-E (t(98) = −12.23, −18.42, and −26.76, respectively; all p < 0.001). Panel F was analyzed using the Mann-Whitney U test (U = 1585.5, z = 2.51, p = 0.012). RCT: root canal therapy; INR: Indian rupee

## Discussion

The principal findings indicate that both RCT-restored teeth and single implant-supported crowns achieved high survival and clinical success. RCT demonstrated 100% survival compared with 96% for implants, although the difference was not statistically significant. Radiographic success was numerically higher with implants, whereas RCT was associated with significantly fewer biological complications, fewer visits, shorter treatment duration, lower cost, and higher satisfaction.

These results are consistent with a systematic review reporting that appropriately treated teeth and single implants show broadly comparable survival, with patient-reported outcomes remaining high across both modalities and better oral health-related quality of life associated with endodontic treatment; that review also emphasized that survival alone is insufficient to guide treatment selection [2]. A randomized controlled trial comparing endodontic retreatment of teeth with an uncertain prognosis against extraction and single-implant replacement similarly found comparable medium-term success, with shorter completion time and better soft-tissue esthetics for the endodontic approach and substantially higher cost for implants-findings that align with the complication, treatment-duration, and cost differences observed here [11].

A long-term retrospective analysis reported that endodontically treated teeth and single-tooth implants both provided predictable survival and success in the short term, but that survival and success of endodontic treatment declined more rapidly than those of implants over longer follow-up [7]. This nuance may explain the numerically higher radiographic success recorded for implants in the present series and highlights the influence of case selection, failure definitions, restoration quality, operator experience, and follow-up length on comparative outcomes. Additional retrospective data likewise indicate comparable survival between the two treatments when correctly indicated [8].

The higher frequency of biological complications observed with implants is in keeping with contemporary evidence that peri-implant mucositis and peri-implantitis are common and frequently require additional interdisciplinary management [9]. Patient satisfaction was favorable for both treatments but significantly higher after RCT, consistent with reports of high patient-reported outcomes and a general patient preference for preserving natural teeth whenever feasible [2]. The lower cost, fewer visits, and shorter duration associated with RCT are supported by economic evaluations showing that nonsurgical root canal treatment is more cost-effective than single-tooth implants [10].

Several sources of bias must temper this interpretation. Because treatment allocation was determined by clinical indications and patient and provider decision-making rather than randomization, confounding by indication is possible. Teeth selected for RCT may have differed systematically from those selected for extraction and implant rehabilitation with respect to restorability, remaining tooth structure, periodontal status, periapical pathology, bone availability, esthetic considerations, and patient preference. Although baseline characteristics were compared, periapical lesions were significantly more frequent in the RCT group, and residual and unmeasured confounding cannot be excluded. The observed differences therefore represent associations within this selected retrospective cohort and cannot be attributed to treatment modality alone.

Treatment selection should accordingly incorporate the remaining tooth structure, ferrule presence, quality of the coronal seal and definitive restoration, periodontal support, and periapical status, together with bone and soft-tissue availability, systemic health, smoking, esthetic demands, finances, and patient preference [1,3,4]. The quality of the final restoration is a key determinant of endodontic success, and vertical root fracture remains an important failure mode for root-filled teeth that must be considered during case selection [4,6]. Evaluating long-term restorative durability and distinct failure modes remains essential when planning definitive coronal reconstructions for compromised dentition [new reference [13]. Consequently, differences in the frequencies of specific biological or technical complications could not be evaluated separately. Future multicenter prospective studies should use standardized outcome definitions, longer follow-up, cost-effectiveness analysis, validated quality-of-life instruments, and multivariable or propensity-adjusted comparisons.

Strengths

The strengths of this study include the comparative evaluation of multiple clinically relevant and patient-centered outcomes, including treatment survival, clinical and radiographic success, biological and technical complications, treatment burden, direct treatment cost, and patient satisfaction. The use of equally sized treatment groups allowed direct comparison across these outcomes. The study also incorporated both clinical and economic measures, providing a broader assessment of treatment selection than survival outcomes alone.

Limitations

Several limitations should be considered when interpreting these findings. The retrospective, nonrandomized design introduces the possibility of selection bias and confounding by indication, particularly given the baseline imbalance in periapical disease between the treatment groups. Selection bias may also have been introduced because records with incomplete information were excluded from the relevant analyses, and the characteristics of excluded records could not be compared with those included. The sample comprised 100 available eligible records without an a priori sample-size calculation and may therefore have had limited statistical power to detect smaller between-group differences, with some estimates showing relatively wide confidence intervals. The small number of outcome events, particularly biological complications, also limited the ability to perform more detailed subgroup or adjusted analyses. Treatment and restoration protocols were heterogeneous, including variations in instrumentation, restorative approaches, and immediate versus delayed implant placement, and the distribution of specific protocols was not consistently available from the retrospective records. The specific criteria underlying prognosis classification could not be independently verified retrospectively. Radiographic outcomes were assessed using treatment-specific criteria that were not directly equivalent between RCT and implant treatment, and a validated radiographic scoring system was not applied; therefore, the between-group comparison of radiographic success should be interpreted cautiously. Radiographic assessment was retrospective and did not include examiner calibration, blinding, or formal assessment of inter- or intra-examiner reliability. Patient satisfaction was retrospectively extracted from clinical records, and its original validation, timing of administration, and completeness could not be independently verified. Biological and technical complications were analyzed as predefined composite outcomes because individual component events were not consistently available. The single-center setting may limit generalizability, while the relatively limited and variable follow-up restricts inference regarding long-term survival and late complications. Residual and unmeasured confounding cannot therefore be excluded.

## Conclusions

In this retrospective single-center cohort of 100 eligible records with relatively limited and variable follow-up, root canal therapy followed by definitive restoration was associated with fewer biological complications, fewer treatment visits, shorter treatment duration, lower direct treatment cost, and higher patient satisfaction, while treatment survival and clinical success were comparable between the groups. Although radiographic success was numerically higher in the implant group, this difference was not statistically significant and should be interpreted cautiously because treatment-specific radiographic criteria were used. Given the nonrandomized design, modest sample size, baseline differences between groups, potential selection bias, and limited follow-up, these findings should be interpreted as associations within the study population and should not be considered evidence of superiority of RCT in routine clinical practice. These findings may inform individualized treatment decisions when a compromised tooth remains predictably restorable, while dental implant treatment remains an appropriate alternative for non-restorable teeth or those with an unfavorable prognosis. Larger prospective multicenter studies with standardized outcome definitions, longer follow-up, and validated patient-reported outcome measures are needed to determine the comparative long-term effectiveness and cost-effectiveness of these treatment approaches.
